# Embracing uncertainty, managing complexity: applying complexity thinking principles to transformation efforts in healthcare systems

**DOI:** 10.1186/s12913-018-2994-0

**Published:** 2018-03-21

**Authors:** Sobia Khan, Ashley Vandermorris, John Shepherd, James W. Begun, Holly Jordan Lanham, Mary Uhl-Bien, Whitney Berta

**Affiliations:** 1 University of Toronto, Toronto, ON Canada; 2 Li Ka Shing Knowledge Institute, St. Michael’s Hospital, Toronto, ON Canada; 3 Division of Adolescent Medicine, Hospital for Sick Children, Toronto, ON Canada; 4 Division of Health Policy and Management, School of Public Health, University of Minnesota, Minneapolis, MN USA; 5 Department of Medicine, University of Texas Health Science Center, San Antonio, TX USA; 6 Veterans Evidence-Based Research Dissemination Implementation Center (VERDICT), South Texas Veterans Health Care System, San Antonio, TX USA; 7 McCombs School of Business, University of Texas at Austin, Austin, TX USA; 8 Neeley School of Business, Texas Christian University, Forth Worth, TX USA

**Keywords:** Complexity, Complex adaptive systems, Systems thinking, Health systems

## Abstract

**Background:**

Complexity thinking is increasingly being embraced in healthcare, which is often described as a complex adaptive system (CAS). Applying CAS to healthcare as an explanatory model for understanding the nature of the system, and to stimulate changes and transformations within the system, is valuable.

**Main text:**

A seminar series on systems and complexity thinking hosted at the University of Toronto in 2016 offered a number of insights on applications of CAS perspectives to healthcare that we explore here. We synthesized topics from this series into a set of six insights on how complexity thinking fosters a deeper understanding of accepted ideas in healthcare, applications of CAS to actors within the system, and paradoxes in applications of complexity thinking that may require further debate: 1) a complexity lens helps us better understand the nebulous term “context”; 2) concepts of CAS may be applied differently when actors are cognizant of the system in which they operate; 3) actor responses to uncertainty within a CAS is a mechanism for emergent and intentional adaptation; 4) acknowledging complexity supports patient-centred intersectional approaches to patient care; 5) complexity perspectives can support ways that leaders manage change (and transformation) in healthcare; and 6) complexity demands different ways of implementing ideas and assessing the system. To enhance our exploration of key insights, we augmented the knowledge gleaned from the series with key articles on complexity in the literature.

**Conclusions:**

Ultimately, complexity thinking acknowledges the “messiness” that we seek to control in healthcare and encourages us to embrace it. This means seeing challenges as opportunities for adaptation, stimulating innovative solutions to ensure positive adaptation, leveraging the social system to enable ideas to emerge and spread across the system, and even more important, acknowledging that these adaptive actions are part of system behaviour just as much as periods of stability are. By embracing uncertainty and adapting innovatively, complexity thinking enables system actors to engage meaningfully and comfortably in healthcare system transformation.

## Background

Complexity thinking is increasingly being embraced in healthcare; this is driven by an acknowledgement that healthcare is complex [1] and thus warrants complex responses [2] rather than traditionally reductionist solutions. Complexity approaches have a rich history rooted in multiple disciplines [3], and are premised on the evolution and transformation of systems [3, 4]. Moreover, complexity approaches align with accepted ideas and philosophies in health care (for example, distributed leadership and patient-centeredness). Understanding the inherent complexity of transforming human systems is particularly important now; healthcare is in an age of transformation, where the convergence of multiple system pressures such as unprecedented life expectancies [5], increasing incidence and prevalence of chronic diseases [6], and globalization of infectious diseases [7] are testing the resilience of health systems worldwide. Complexity thinking can provide guidance to system actors (i.e., people, organizations and other components that comprise the system) on how to respond to these pressures and to transform healthcare systems in innovative, collaborative, and action-oriented ways. In this paper, we aim to clarify insights from complexity thinking that may help us understand how to manage complexity in health care.

### The relevance of complexity thinking to healthcare

Healthcare (including patient self-management, delivery of health services and programs for acute and chronic conditions, public health, and long-term care) is often described specifically as a complex adaptive system (CAS) [3], a term that requires unpacking. A “system” is created when the level of *connectivity* between actors fosters interdependence on one other; in a system, the action of one actor can have broader implications for other connected actors [3]. These interdependencies determine the nature, scope and size of the system [3]. A system becomes “complex” because the interdependencies that define the system also render it highly dynamic, oscillating between periods of stability and chaos [3]. The healthcare system is hierarchical and comprises multiple (micro, meso and macro) levels that are nested or embedded within one another [3], which increases dynamism and therefore, complexity. A single patient-physician interaction may occur within an organization that consists of its own wider system of interdependencies that enforces or constrains how the organization operates; the organization may have multiple interdependencies with other organizations to secure operational necessities (such as resources) and deliver services in a network of patient care; this network of organizations exists in a broader system that sets priorities and policies, and allocates resources that dictate how patient health is managed within these multiple systems. The dynamic nature of complex systems imbues them with a Bayesian quality in which probabilities of outcomes are perpetually updated as new information is continuously introduced, and implies that a certain amount of “irreducible uncertainty” will persist within the system [8]; therefore a complex system is characterized by a high degree of uncertainty and a low level of agreement between system actors about the causes of system pressures and the potential solutions to alleviate those pressures [9–11]. This irreducible uncertainty contributes to the non-linearity between cause and effect – that inability to attribute outcomes to actions that often plagues healthcare.

The reactive potential of a system to change in response to system pressures is what is known as *adaptability*, and what makes a complex system an adaptive one. Our growing interest in CAS stems from our growing interest in healthcare system transformation. Complex systems naturally transform through adaptation – indeed, adaptability is a measure of a system’s potential to change or transform. In complexity science, system actors self-organize through local interactions, which leads to the emergence of new patterns of system norms and behaviours. Self-organization, then, seems to be a characteristic of the system that facilitates adaptation. Therefore, in any current or future restructuring of our healthcare system, we aim to position our systems such that adaptability can contribute to positive system transformation.

The paper was born out of a seminar series that took place in April 2016 at the University of Toronto that focussed on theory and practice in complexity and systems thinking and their application to healthcare [9, 11–14]. The series engaged experts in the areas of complexity science and practice, and offered a diverse set of topics including assessing complexity in global health research; complexity leadership; complexity in clinical care; and the theoretical underpinnings and origin of complexity science. As a whole, the series offered a number of insights on applications of CAS purviews to healthcare that we explore here. In synthesizing the observations made by these experts, we augmented these insights with others gained through literature focussing on the intersection of complexity and systems thinking concepts with healthcare. Some CAS scholars suggest that CAS concepts can be instructive for system transformation efforts [1] while others contend that application of CAS concepts to human social systems is entirely inappropriate [15]. Throughout this paper then, in addition to discussing insights afforded by applying CAS concepts to healthcare system transformation, we also discuss this paradox with respect to applying CAS concepts to human social systems.

## Key insights on the application of complexity science to health care

### Insight 1: A complexity lens helps us better understand the nebulous term “context”

Complexity in healthcare was acknowledged long before complexity thinking made its influence explicit. Deviations from expected outcomes are often ascribed to “context”; that the relations we assume we know between outcomes and actions are perturbed by a multitude of known and unknown factors. The prevalent discussion about the role of context in healthcare is in itself is an acknowledgement of complexity [9]. Making complexity thinking explicit challenges us to go one step further; to think beyond the discrete influence of particular contextual factors and to consider the connectedness of those factors, or perhaps, the *systemness* of the system. In 2009, the World Health Organization acknowledged that context is in fact informed by a wider dynamic of system interactions [16]. Foster-Fishman et al. assert that studying the system rather than context “better captures the ecological and social change emphasis of the field than context” [17]. In their framework for systems change, they emphasize approaches to assessing system context and the multidirectional role between context, system actors, and interventions by analyzing interdependencies and patterns of system behaviour [17]. Current approaches to analyzing context involve assessing contextual factors and their distinct influence on actors and interventions without explicit reference to multi-way interdependencies. Using a complexity approach to understand context as a system phenomenon may help perpetuate better understanding and analysis of this fuzzy concept.

### Insight 2: Concepts of CAS may be applied differently when actors are cognizant of the system in which they operate

Healthcare is a human system, the functionality of which relies on human cognition and sociality [18]. CAS principles were originally derived from observations of natural systems (such as ecosystems), whose system actors are unaware of the system(s) in which they operate - indeed, systems are a human social construct. In natural systems, adaptations occur as emergent responses to system pressures without system actors being cognizant of the system’s adaptability. In human systems, adaptation may be similarly emergent through slow, incremental change, or may occur rapidly when there is a substantial perturbation of the system. But unlike natural systems, some adaptation must be concerted or intentional in human systems, given that human systems are characterized by entrenched social and political structures that are designed to impose order on a complex and chaotic world.

Applying complexity thinking to the notion of enhancing system adaptability in a concerted and intentional way is admittedly paradoxical given the conceptualization of complex adaptive systems as unplanned, unmanaged, non-linear and non-hierarchical emergence-and-feedback systems. For example, self-organization is premised on a lack of cognition about the system’s behaviour, but in human systems, intentional efforts to change and transform are commonplace. The simple rules of a system are also premised on a lack of awareness about what those simple rules are; nonetheless, some CAS scholars suggest that a form of system self-organization can be guided by “developing” simple rules, such as coordination and cooperation [19], to make sense of (and perhaps try to influence) the system’s behaviour. An example of this in healthcare is the prioritization of integrated care, where coordinated and strategic efforts are being imposed to transform existing health service delivery structures and foster emergent interorganizational and cross-sector collaborations across the system [20]. So while the tenets of CAS still apply, there are cases in which system transformation has both emergent and intentional properties in human systems. The implication of intentional transformation efforts is that system actors are conscious of the system in which they reside and acknowledge it as being complex; this introduces an element of reflexivity, where human perceptions and actions in response to system dynamics become themselves causes of these dynamics. At the same time, there are elements of human systems of which actors will never be full conscious, where emergent adaptations occur through unintentional actions, and where unintended consequences result from intentional efforts. In this way, there remains a duality where actors in human systems may be both cognizant, and not cognizant, of the nature of the CAS.

Cognition in CAS is not well-described or well-accounted for in the complexity literature, therefore the conceptual and practical implications for healthcare systems are not well understood. John Paley brought attention to this in his perspective paper on complexity theory [15]; he contends that complexity (and CAS) functions to explain system behaviour, but when cognition is introduced, explanatory variables such as self-organization are no longer relevant. Since the notion of cognition underlies the tents of CAS and complexity thinking in general, further debate is required in this area to understand applications of CAS to human systems.

### Insight 3: Actor responses to uncertainty within a CAS is a mechanism for emergent and intentional adaptation

As described above, pressures affecting a complex system stem from and result in uncertainty. We argue that while traditional responses of system actors aim to reduce uncertainty, embracing complexity requires embracing a level of irreducible uncertainty - given that uncertainty elimination cannot exist in a complex system with no finite and simple solutions [21]. In healthcare systems, uncertainty is prevalent at multiple levels and is faced by multiple system actors including patients, frontline clinicians, managers and leaders, policymakers, and researchers. Managing uncertainty may require minimizing the emphasis on standardized processes (for example, checklists), and encouraging alternative approaches to quality management and improvement that enable exploration of multiple potential solutions [21]. Bar-Yam [22] recommends classifying problems according to their complexity, and introducing solutions accordingly. For example, in situations where a low level of uncertainty exists about the solution to the problem, more standardization can be introduced, which may enhance the overall efficiency of the system. Where a higher level of uncertainty exists about the solution to a problem, activities that encourage innovation production, relationship building and “trial-and-error” solutions can be introduced. These approaches are described in the sections below.

### Insight 4: Acknowledging complexity supports patient-centred and intersectional approaches to patient care

William Osler famously stated “Listen to your patient, he is telling you the diagnosis” [23]. Patient-centred care (a term popular in clinical settings) and intersectionality and health (a term popular in social, political and public health spheres) are widely accepted ways of viewing care. However, these views are not necessarily applied in approaches to care. Complexity thinking functions to support a rationale for patient centredness and intersectionality approaches, and can provide methods of applying such approaches. Reconsidered from a complexity stance, Osler’s suggestion hints at the insights clinicians may gain by viewing the patient as an embodiment of embedded complex systems (through biological and disease mechanisms), and as an individual whose health and healthcare are shaped by the embeddedness of other complex systems (e.g., social support, education, access to resources and services, and larger social and political structures). At the same time, having patients understand the interdependencies affecting their health may ensure that they are vocal in communicating and questioning the role of these interdependencies in a clinical encounter. The involvement of patient values and voices in decision-making aligns with a complexity perspective that underscores the tension between uncertainty reduction (i.e., making decisions based on their likelihood of successfully mitigating uncertainty) and uncertainty absorption (i.e., acknowledging the extent of interdependencies and the numerous potential “solutions”, with no one solution necessarily being the “right” solution; recognizing that uncertainty reduction is potentially unachievable and is no longer the de facto priority). Often, in an attempt to reduce uncertainty, clinicians who take on the traditional role of sole decision maker may (unintentionally) minimize the numerous interdependencies affecting a patient’s health; the patient-centred approach may result in greater uncertainty absorption than reduction, and can allow for a consideration of solutions that match the complexity of the condition.

Potential methods of embedding patient centredness and intersectionality into clinical processes include a system level commitment to enhanced patient education that stresses the interdependencies that affect health. Frontline clinicians would benefit from training that would enhance comfort with absorbing the residual uncertainty that defines complex systems. Participating in shared sense-making is one approach to uncertainty absorption and can allow front-line clinicians to thrive within a CAS [24]. Shared sense-making, described as “a diagnostic process directed at constructing plausible interpretations of ambiguous cues that are sufficient to sustain action” [24] provides front-line clinicians with a platform through which to process uncertainty and unpredictability and to discern an appropriate course of action in the midst of an ever-evolving landscape. Valuing relationships including the protection of time and space for clinical dialogue between patients and providers, as well as between collaborating providers, is required to apply patient-centred and intersectional approaches [23]. At a health system level this can inform - and potentially radically alter - models of care [25].

### Insight 5: Complexity perspectives can support ways that leaders manage change (and transformation) in healthcare

Leadership from a complexity perspective is not predicated on hierarchy and formal roles, but ascribed to individuals who contribute ideas and take action [26]. It also tends to be viewed as an emergent phenomenon that is observable as a result of the interaction among dynamic agents in a CAS [27]. Begun states that “in a complex organizational setting, leadership tasks (for example, finding direction, building commitment, and overcoming challenges) are accomplished by emergent, relational dialogue among diverse individuals” [28, 29]. This definition aligns with notions of distributed leadership, where leadership is not role-based but action-based, and where leadership is a social process amongst a group rather than the action of an individual [30].

However, the reality of healthcare systems and organizations is that many are still governed using traditional conceptualizations of leadership that promote what is essentially a top-down approach in which leaders in formal positions impose order and control in an attempt to align system actors with the vision of the organizational system [11]. Where traditional leaders strive to eliminate chaos and reduce uncertainty, complexity leaders thrive in chaos and absorb uncertainty.

Although there appears to be tension between these two leadership types, Uhl-Bien [31] contends that they can co-exist within healthcare organizations. Similar to aligning responses to uncertainty with level of uncertainty, different types of leadership may be required for different objectives and functions of the organization. Traditional leadership may be warranted when institutionalization and control is demanded. Complexity leadership may be required when a system or organization faces complex challenges. Oftentimes, novel ideas emerge in response to pressures or challenges; these entrepreneurial efforts ensure that the system or organization remain adaptive. Complexity leaders stimulate innovation and ensure that there is adequate space, time and resources to allow these innovations to materialize. They support ideas and curate them for formal (traditional) leaders, who are capable of institutionalizing innovations through their top-down control mechanism. In this way, Uhl-Bien imagines a system that can embrace complexity through the actions of leaders who operate in the “adaptive spaces” between the institutional and entrepreneurial arms of an organization, and who work in conjunction with traditional leaders to formalize change [11].

However in many cases (perhaps due to increasing acknowledgement of complexity and an established field of leadership research that supports transformative and adaptive leadership styles), formal leaders (defined here as an institutionalized leadership position) would benefit from approaching leadership from a complexity perspective. Those seeking to cultivate innovation in the face of uncertainty must recognize social dynamics as foundational to developing adaptive responses to system pressures. Complexity-inspired formal leaders are encouraged to foster relationships and collaborative communication, and promote mindful (open to new explanations for ordinary events) and reciprocal (iterative) learning [32]. Such interactions will in turn facilitate shared sense-making, which is fundamental to supporting adaptation. Begun and Malcolm argue that “leadership to meet complex challenges involves working on shared sense-making, exploration of strategic options through action and learning from those actions, and altering and increasing connections among individuals, teams, departments and stakeholders” [27]. Complexity-inspired formal leadership demands humility and patience, suppressing the instinct to troubleshoot while exploring several approaches and slowly shifting time and attention to solutions that seem to work [1, 8]. It draws on the notion of “sponsoring”, in which leaders seek action within the system, connect actors to catalyze relationships, and plant seeds for emergent ideas and innovations [11]. Begun and Malcolm state “[t]he most important competency for complexity-inspired leaders may be asking questions rather than giving answers” [27].

In organizations, leaders may also be managers who are often directly charged with operationalizing change efforts, which, in the current climate of healthcare, are typically directed, intentional changes. Within a CAS, such a responsibility demands that managers - even more acutely than strategic leaders perhaps - cultivate exploration and foster innovation. Managing CAS requires an enhanced attentiveness to the nature and interactions of system actors to determine how uncertainty can be harnessed into positive adaptation and innovative practices. It is recommended that managers promote exploration as a response to uncertainty, while also encouraging “dissent and diversity” [28, 33]. These ideas are consistent with Uhl-Bien’s concept of adaptive space, described above [11], and are aligned with the concept of “positive deviance”, a change management technique that endeavours to support the identification of “sustainable solutions hidden within plain sight” by promoting the emergence of group learning and adaption [27]. For example, in order to improve quality and team performance in a CAS, Bar-Yam suggests empowering group competitions that let teams work locally and non-prescriptively to stimulate innovation using an evolutionary dynamic: team competition (e.g., posting infection rates by ward) [12]. Begun and Thygeson note that in order to encourage diversity and exploration amongst teams, managers must ensure that a system has effective communication processes to allow all voices to be heard, but must “curate the work of the group” to ensure that quality standards are adhered to [28]. Complexity thinking suggests acknowledging rather than seeking to resolve the tension between these demands. One tactic to working within this tension may be the “act-then-look” approach [8], which suggests that because CAS are inherently unpredictable and in flux, learning which informs innovation may be best achieved by acting first and then seeking feedback [28]. This approach is similar to the plan-do-study-act approach [34]; what a complexity perspective adds is creating conditions around how novel ideas emerge. Managers comfortable working within CAS will seek to manage initial conditions, monitor for emergence of innovations [33], and direct innovations to benefit the adaptability of the system.

### Insight 6: Complexity demands different ways of implementing ideas and assessing the system

Policy landscapes are recognized as CAS, characterized by uncertainty and emergent properties and behaviours that are unpredictable, subject to the influence of often unrecognized actors, and shaped by co-adaptive dynamics. Furthermore, policymaking is always intentional although, consistent with CAS, implemented policies do not always produce the intended result, and the potential for policy resistance, defined as “the tendency for interventions to be defeated by the system’s response to the intervention itself”, often dominates [35]. Therefore, it becomes difficult to apply theory positing linear mechanisms of action to policy-making and policy analysis; complexity approaches may be better suited. Weick argues that given the inherent unpredictability of a CAS, policymakers should embrace an iterative “act-then-look” approach, as described above, at a small scale first to determine which actions merit system level spread [36]. Through a complexity lens, theory does not drive policy creation or adequately predict outcomes. Rather, it becomes a tool for reflection and sense-making, “the practice of creating an intellectual coherence from emerging conditions” [37] – in essence, learning in an iterative way for more optimal policy implementation.

Complexity thinking also carries implications for the nature of scientific inquiry; some argue that complexity science is a “new scientific ontology” [37], perhaps in reference to the complexity perspective that values the principles of complex systems and a commitment to research approaches that can address these principles – although this is arguably illustrative of the paradigm of pragmatism. A complexity science worldview implies a particular manner of framing questions and a specific quality to the answers being sought. It influences how the researcher is positioned within his or her inquiry and how that researcher engages with the subject of interest. A complexity framework embraces uncertainty in the research process, since this is recognized as a critical condition for creating the space for important social exchange and for allowing research questions to emerge and re-emerge [8]. Espousing a complexity worldview will inherently define the shape, course, and implications of the research endeavour. Furthermore, researchers who embrace complexity science as a paradigm will be better positioned to aid those working in the healthcare system to introduce intentional change within a CAS. Moving beyond the philosophical, a complexity science paradigm has pragmatic implications for researchers. Studying CAS requires a set of research tools that facilitates an examination and exploration of phenomena that are dynamic, non-linear, co-adaptive and emergent. Traditional gold-standard approaches to research, such as randomized controlled trials, have been proven mathematically to be limited in their capacity to generate sufficient evidence to capture all potential conditions and permutations [38], and aim to minimize uncertainty rather than explore potential sources of irreducible uncertainty. A new repertoire of complexity-informed research “tools” that account for the properties of CAS have been proposed. These include such approaches as system dynamics, discrete event simulation, agent-based modeling, fuzzy set qualitative comparative analysis, and social network analysis [27], in addition to standard research approaches.

## Discussion

In this paper, we discuss insights gained from a complexity science seminar series at the University of Toronto. We describe how CAS principles underlie accepted health system philosophies and initiatives. We also describe how actors within the healthcare system (patients, frontline clinicians, managers and leaders, policymakers, and researchers) can apply CAS principles to both emergent and intentional change efforts. Complexity thinking is highly relevant to understanding and transforming healthcare systems, which are inherently complex and changing. Multiple insights from complexity thinking can help shape how we perceive the system and the role of actors within the system. Complexity thinking also supports existing notions we have about the nature of a given system, but offers additional perspectives on how these ideas might be enacted or assessed. The six insights we highlight from our expert seminar series were ideas that we believed would contribute to additional understanding about accepted ideas that already acknowledge complexity (context and patient-centredness), that we think merit further debate in order to further understand applications of complexity (cognition), or that carry practical implications for how complexity may be considered in practice (implications of acknowledging uncertainty, complexity for leaders, and methods of assessing complexity).

A common thread throughout these points is the notion of responses to uncertainty (Insight 3). System complexity begets uncertainty, and levels of uncertainty in CAS can sometimes be extreme. Managing this uncertainty is what system actors are trying to accomplish when undergoing system adaptation (or transformation), and actors can take on specific behaviours, actions and roles to facilitate intentional adaptation (in addition to emergent adaptation), which is a unique characteristic of CAS in human systems. We note that although these actions can ultimately lead to system adaptation, the multi-level and networked nature of a complex system renders the outcomes of these actions difficult to predict. In other words, the outcomes of responses to uncertainty are uncertain in themselves – which makes the exercise of *embracing* complexity rather than fighting it all the more worthwhile.

Adopting complexity thinking in healthcare systems requires system actors to work against many of the deeply engrained structural and social norms that prevail. Commonly practiced approaches to delivering and understanding healthcare is driven by reductive solutions and as much standardization as possible. A CAS-informed approach to transforming healthcare systems requires a connectionist (and potentially bottom-up) perspective, where knowledge is dynamic and diffuse and quality improvement requires variability in response to local conditions. Experts suggest introducing complexity thinking to existing structures by “reframing complexity” to other actors working in the system – in other words, deemphasizing the abstract concepts of complexity thinking that are so challenging to apply to specific systems, while encouraging system actors to “complexify” their own thinking by raising awareness of the systemness of their systems as it relates to such elements as their work, behaviour, and health.

While we highlight several insights afforded by applying complexity thinking to concerted attempts to enhance system adaptability, we also acknowledge the paradox of applying concepts intended to describe natural systems to socially constructed human systems. For example, the issue of setting artificial, socially constructed system boundaries is well-known and well-discussed. Approaches to examining CAS involve acknowledging the system’s complexity while necessarily reducing it to the extent that we can study it – as such, we impose socially-constructed boundaries within a larger system, when no real boundaries exist [14], in order to be able to examine a smaller system that is ostensibly less complex. The issue of cognition in complexity factors is important to our understanding of which factors apply to intentional change and transformation in human systems. One limitation we noticed in the application of CAS perspectives in the literature to date is that they have focused on the organization as a complex system, despite the potential to understand larger healthcare systems - i.e.¸ multiple organizational actors and a cadre of independent practitioners not affiliated with organizations - using a CAS perspective. Future applications of CAS could aim to widen the “boundaries” in which the CAS lens is being applied.

## Conclusions

This paper highlights key aspects of complexity thinking that can be applied in practice, and other aspects that may warrant further debate and exploration. Ultimately, complexity thinking acknowledges the “messiness” that we seek to control in healthcare and encourages us to embrace it. This means seeing challenges as opportunities for adaptation, stimulating innovative solutions to ensure positive adaptation, leveraging the social system to enable ideas to emerge and spread across the system, and even more important, acknowledging that these adaptive actions are part of system behaviour just as much as periods of stability are. By embracing uncertainty and adapting innovatively, complexity thinking enables system actors to engage meaningfully and comfortably in healthcare system transformation.

## Abbreviation

CAS: Complex adaptive system
